# When Tears Signal Vasculitis: Bilateral Dacryoadenitis as the Initial Manifestation of Granulomatosis with Polyangiitis—Case Report

**DOI:** 10.3390/reports9010025

**Published:** 2026-01-14

**Authors:** Sylvia Kutsarova, Tsvetoslav Georgiev, Miroslava Benkova-Petrova, Aleksandar Petrov, Hristo Popov

**Affiliations:** 1 Faculty of Medicine, Medical University of Varna, 55 Marin Drinov St., 9002 Varna, Bulgaria; 2 Clinic of Rheumatology, University Hospital “St. Marina” EAD, 1 Hristo Smirnenski Blvd., 9010 Varna, Bulgaria; 3 Clinic of Nephrology, University Hospital “St. Marina” EAD, 1 Hristo Smirnenski Blvd., 9010 Varna, Bulgaria; 4 Clinic of General and Clinical Pathology, University Hospital “St. Marina” EAD, 1 Hristo Smirnenski Blvd., 9010 Varna, Bulgaria

**Keywords:** granulomatosis with polyangiitis, ANCA-associated vasculitis, dacryoadenitis, orbital inflammation, PR3-ANCA, crescentic glomerulonephritis, pauci-immune GN, early diagnosis, renal involvement

## Abstract

**Background and Clinical Significance:** Granulomatosis with polyangiitis (GPA) is an ANCA-associated vasculitis that often affects the respiratory tract and kidneys, while ocular involvement is less common and may delay diagnosis. Bilateral dacryoadenitis as an initial manifestation is particularly uncommon and can obscure early recognition. **Case Presentation:** A 24-year-old woman presented with recurrent epistaxis, headaches, and progressive bilateral eyelid swelling. MRI showed enlarged lacrimal glands consistent with granulomatous dacryoadenitis. Over the following weeks, she developed systemic symptoms and rapidly progressive renal impairment. Serology revealed positive c-ANCA and anti-PR3 antibodies, and HRCT demonstrated pulmonary nodules and ground-glass opacities. Renal biopsy confirmed necrotizing pauci-immune crescentic glomerulonephritis. Despite treatment with glucocorticoids, cyclophosphamide, and rituximab, renal recovery was incomplete, necessitating hemodialysis. **Conclusions:** This case illustrates bilateral dacryoadenitis as an early sign of GPA and emphasizes the need for prompt ANCA testing and renal evaluation. Early recognition is crucial to prevent irreversible kidney damage.

## 1. Introduction and Clinical Significance

Granulomatosis with polyangiitis (GPA), formerly known as Wegener’s granulomatosis, is a necrotizing vasculitis primarily affecting small- to medium-sized vessels, characterized by granulomatous inflammation and a strong association with anti-neutrophil cytoplasmic antibodies (ANCAs), particularly those directed against proteinase 3 (PR3) [1,2]. The disease most commonly involves the upper and lower respiratory tracts and kidneys, forming the classical triad of GPA; however, atypical presentations involving the orbit, skin, or nervous system may precede systemic manifestations, delaying diagnosis and treatment [3,4,5].

Ocular and orbital involvement occurs in approximately 30–45% of patients and may represent the initial manifestation of the disease. Clinical presentations range from episcleritis and scleritis to orbital pseudotumor and dacryoadenitis, often mimicking other inflammatory or infectious conditions [6,7,8]. These localized symptoms, if unrecognized, can progress to life-threatening systemic vasculitis, including pulmonary hemorrhage and rapidly progressive glomerulonephritis (RPGN) [9,10]. Early recognition of such atypical phenotypes is therefore crucial for timely diagnosis, histopathologic confirmation, and the initiation of immunosuppressive therapy [11].

Recent advances in the understanding of autoimmune vasculitis have highlighted the need for multidisciplinary management integrating rheumatology, nephrology, and ophthalmology [12,13]. Contemporary therapeutic strategies include glucocorticoids in combination with cyclophosphamide or rituximab for remission induction, followed by maintenance therapy to prevent relapses [14,15]. Nevertheless, delayed recognition—especially in cases with initial ocular or sinonasal involvement—remains a key determinant of irreversible renal damage and chronicity [16,17]. This report describes a patient with PR3-ANCA-positive GPA initially presenting with bilateral dacryoadenitis and later progressing to pauci-immune crescentic glomerulonephritis, emphasizing the diagnostic challenges of orbital-onset vasculitis.

Isolated dacryoadenitis as an initial presentation of GPA has been documented only in a few case reports. For example, Lopes Caçola et al. described a young woman who presented with bilateral dacryoadenitis as the first manifestation of orbital-limited GPA [18]. Another recent report detailed a patient with GPA who had bilateral lacrimal gland enlargement along with pancreatic involvement [19]. These cases highlight the rarity of lacrimal gland involvement as the first manifestation of GPA and underscore the importance of considering ANCA-associated vasculitis in the differential diagnosis of refractory orbital inflammation. This report describes a young patient with PR3-ANCA-positive GPA initially presenting with bilateral dacryoadenitis and later progressing to pauci-immune crescentic glomerulonephritis, emphasizing the diagnostic challenges of orbital-onset vasculitis.

## 2. Case Presentation

A 24-year-old woman with no previous medical conditions, autoimmune disease, or family history of vasculitis presented in November 2024 with recurrent epistaxis, nasal obstruction, and persistent frontal headaches. Initially, the symptoms were interpreted as chronic sinusitis and managed with antibiotics and topical corticosteroids, without improvement. Over the following weeks, the patient developed progressive nasal congestion, facial pain, fatigue, and intermittent fever, as well as swelling of the left upper eyelid associated with tearing and discomfort. An ophthalmologic examination documented bilateral eyelid edema, more pronounced on the left, with mild conjunctival injection, preserved extraocular movements, and normal visual acuity. Differential diagnoses included viral dacryoadenitis, orbital pseudotumor, and sarcoidosis. The patient was started on empirical corticosteroid drops without significant effect.

**Figure 1:**
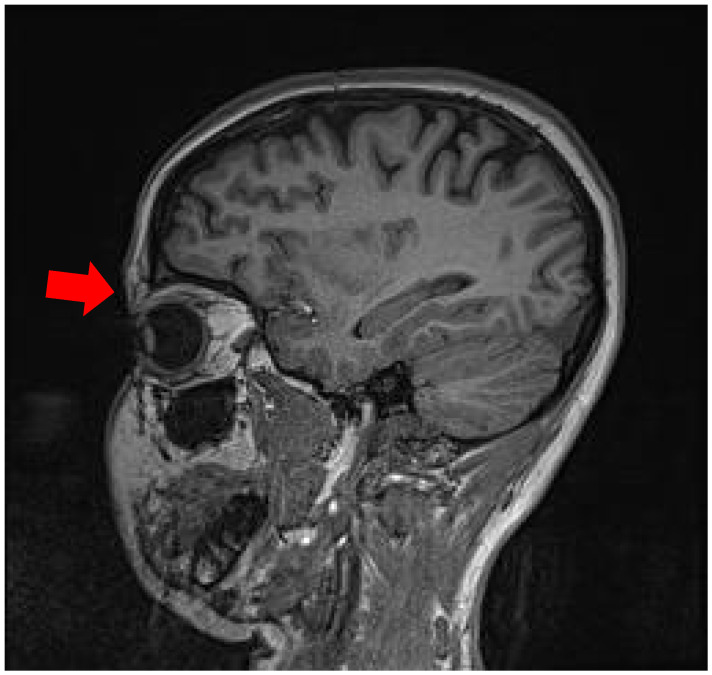
Orbital MRI: Bilateral lacrimal gland enlargement with homogeneous signal and periorbital fat stranding (arrow)—findings consistent with granulomatous dacryoadenitis.

**Figure 2:**
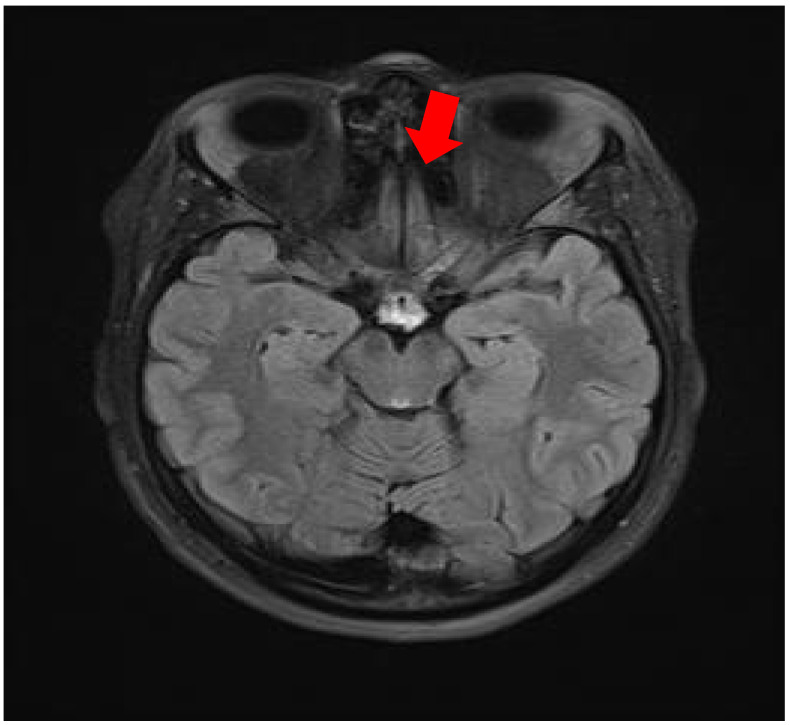
Paranasal sinus MRI: Mucosal thickening in the anterior nasal cavities with partial obstruction of the maxillary ostia (arrow), suggestive of inflammatory/granulomatous involvement.

In January 2025, she was admitted to the Department of Otorhinolaryngology at University Hospital “St. Marina,” Varna, for detailed evaluation. Anterior rhinoscopy revealed markedly narrowed nasal passages with friable mucosa, septal deviation, and hemorrhagic secretions. MRI of the orbits demonstrated bilateral lacrimal gland hypertrophy, homogeneous signal intensity, and periorbital fat stranding, suggesting granulomatous dacryoadenitis. The paranasal sinuses showed mucosal thickening in the anterior nasal cavities and partial obstruction of the maxillary ostia, suggesting inflammatory or granulomatous involvement (Figure 1 and Figure 2).

In the following month, the patient reported generalized weakness, myalgia, arthralgia, and dry cough. She was admitted to the Department of Rheumatology (February 2025) for evaluation of a possible systemic inflammatory disorder. On physical examination, she appeared moderately ill with facial swelling, pale mucosa, and bilateral periorbital edema. No purpura or skin ulcerations were observed. The lungs were clear on auscultation, heart sounds were regular, and mild tenderness was noted in the right costovertebral area. Laboratory findings revealed normocytic normochromic anemia (Hb 116 → 92 g/L), elevated inflammatory markers (CRP 48.8 mg/L, ESR 100 mm/h, fibrinogen 7.86 g/L), and progressive renal impairment—serum creatinine rose from 72 µmol/L to 516 µmol/L and urea from 4.3 to 23.5 mmol/L within two weeks. Urinalysis demonstrated 2+ proteinuria, 3+ hematuria, and numerous red blood cells (2215/µL). The 24-h urinary protein excretion was 2.0 g. Immunological testing revealed positive cytoplasmic ANCA (c-ANCA) by indirect immunofluorescence and anti-proteinase 3 antibodies (anti-PR3 91 U/mL, normal <5) by ELISA. Antibodies against myeloperoxidase (anti-MPO), double-stranded DNA, and antinuclear antibodies (ANA) were negative. Complement levels (C3, C4) were within normal ranges. Rheumatoid factor was modestly elevated (35 IU/mL).

**Figure 3:**
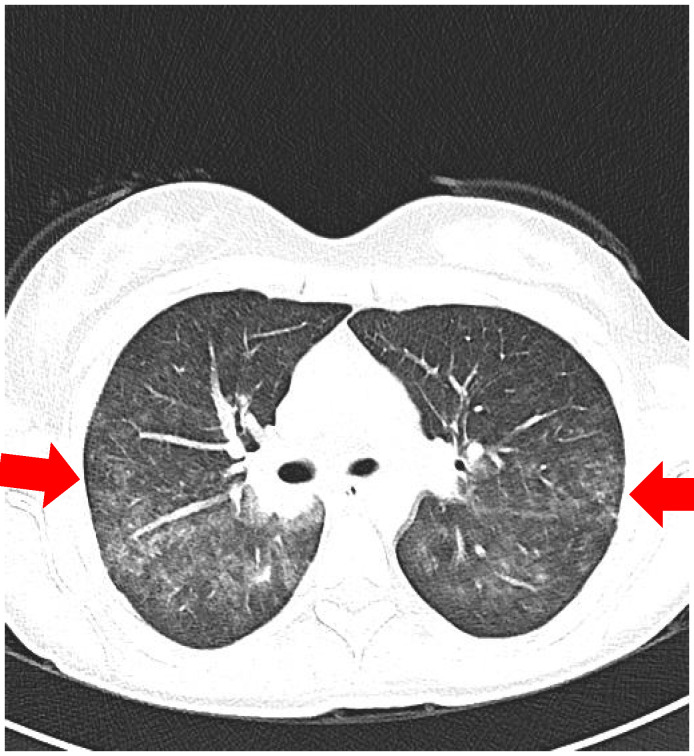
Thoracic HRCT (lung window): Axial image demonstrating bilateral ground-glass opacities with smooth interlobular septal thickening (“crazy-paving”) and multiple peribronchovascular nodules (right > left).

**Figure 4:**
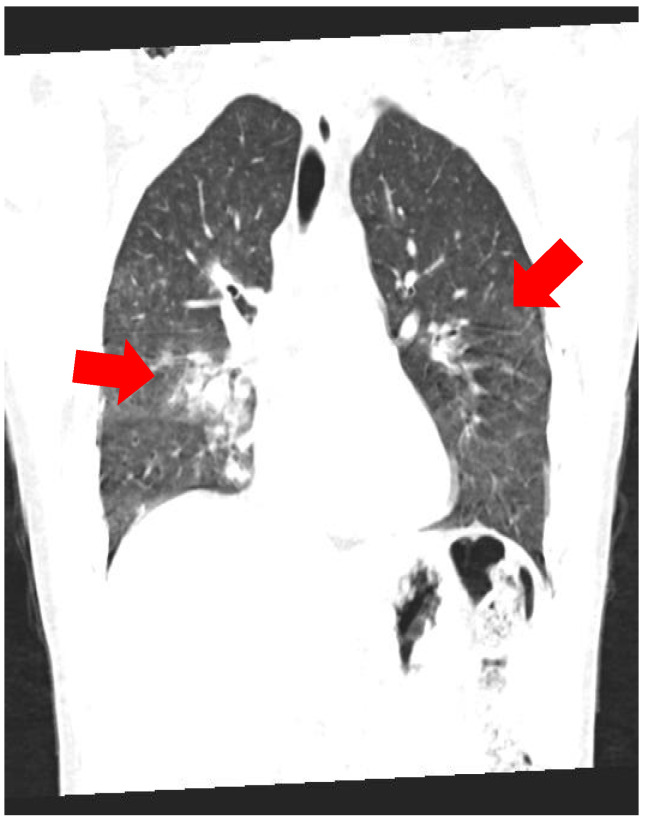
Thoracic HRCT (lung window): Axial image at a different level showing similar changes in both lungs.

A chest X-ray showed a right perihilar infiltrate, while high-resolution CT (HRCT) of the thorax revealed multiple peribronchovascular nodules, diffuse ground-glass opacities, and areas of “crazy-paving” (interlobular septal thickening) in both lungs—features compatible with pulmonary vasculitis or granulomatous inflammation (Figure 3 and Figure 4).

**Figure 5:**
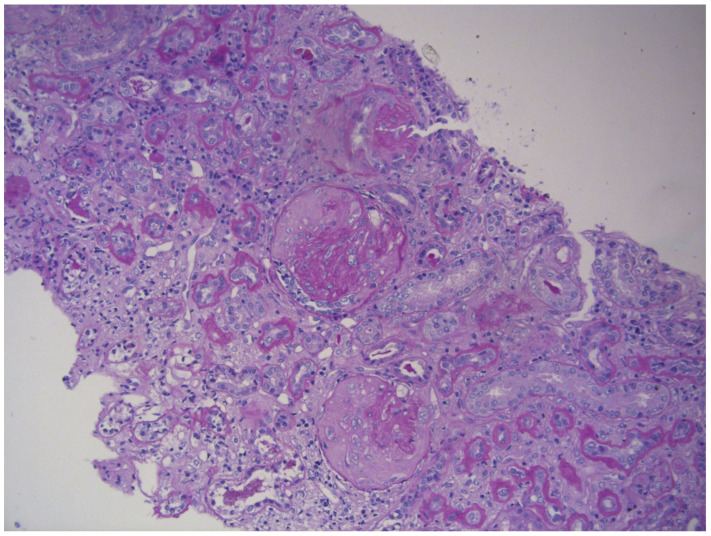
Renal biopsy (light microscopy, PAS ×200): Necrotizing crescentic glomerulonephritis with fibrinoid necrosis (, segmental capillary collapse, and periglomerular fibrosis; >50% tubular atrophy with moderate interstitial fibrosis and focal lymphoid infiltrates.

A percutaneous ultrasound-guided renal biopsy was performed. Light microscopy revealed necrotizing crescentic glomerulonephritis with fibrinoid necrosis, segmental glomerular collapse, and periglomerular fibrosis. Over 50% of tubules exhibited atrophy, accompanied by moderate interstitial fibrosis (26–50%) and focal lymphoid infiltration. Direct immunofluorescence showed only nonspecific deposits of C1q, C3, and IgM, confirming a pauci-immune pattern typical for ANCA-associated vasculitis (Figure 5).

The diagnosis of Granulomatosis with Polyangiitis (GPA) was established according to the 2022 ACR/EULAR classification criteria, supported by the positive c-ANCA/anti-PR3 antibodies and Birmingham Vasculitis Activity Score for Wegener’s Granulomatosis (BVAS/WG) activity score of 7, indicating active systemic vasculitis with renal and ENT involvement (criteria: ENT/orbital involvement and active renal involvement with rapidly progressive kidney dysfunction and active urinary sediment; ACR/EULAR GPA classification components in this patient: PR3-ANCA positivity; sinonasal involvement; pulmonary abnormalities consistent with granulomatous disease/vasculitis; and biopsy-proven pauci-immune necrotizing crescentic glomerulonephritis).

Induction therapy was initiated with pulse methylprednisolone 1 g IV daily for 3 consecutive days, followed by oral prednisone (1 mg/kg/day) with gradual tapering. Cyclophosphamide 1 g IV monthly, administered as six pulses over six months (cumulative dose 6 g), was added as standard induction therapy. Despite a transient improvement in inflammatory markers, renal function continued to decline. Rituximab (1000 mg IV, two doses 14 days apart) was introduced as maintenance therapy after six months due to inadequate response. During this period, hemodialysis was started because of end-stage renal failure. The patient’s general condition improved gradually after immunosuppressive therapy, and systemic inflammation was controlled.

The table below (Table 1) provides an overview of the patient’s key laboratory findings at presentation (February 2025).

## 3. Discussion

GPA represents a heterogeneous autoimmune vasculitis with protean manifestations, in which atypical initial presentations may obscure the diagnosis. Our patient’s clinical evolution—from isolated bilateral dacryoadenitis to renal involvement—illustrates this diagnostic complexity. Orbital inflammation, although well documented in systemic GPA, rarely occurs as the first manifestation and can clinically mimic idiopathic orbital pseudotumor, lymphoma, or sarcoidosis [6,7]. In this case, the chronicity and poor response to topical therapy made viral dacryoadenitis or idiopathic orbital inflammation less likely. Sarcoidosis was also considered, given the lacrimal gland involvement; however, the overall clinical pattern together with ANCA seropositivity and the subsequent demonstration of pauci-immune crescentic glomerulonephritis, supported a diagnosis of GPA. The presence of bilateral lacrimal gland enlargement, recurrent orbital inflammation, and sinonasal involvement should therefore raise clinical suspicion for systemic vasculitis, particularly when accompanied by elevated inflammatory markers and positive ANCA serology.

The renal component of GPA remains the most important determinant of long-term prognosis. Pauci-immune crescentic glomerulonephritis develops in approximately 70–80% of patients and may progress rapidly to end-stage kidney disease if not promptly recognized and treated [8,10]. In our patient, renal biopsy already demonstrated significant chronic changes, including glomerulosclerosis and interstitial fibrosis, consistent with delayed diagnosis. Renal biopsy remains essential not only for diagnostic confirmation but also for assessment of disease chronicity and prognostication [11,12]. Multiple studies have shown that the degree of histologic chronicity at presentation strongly predicts renal outcome and limits the potential for renal recovery despite adequate immunosuppressive therapy [13,14].

Immunosuppressive therapy in GPA aims to induce remission and prevent organ damage. The introduction of rituximab, a monoclonal anti-CD20 antibody, has provided an alternative to cyclophosphamide for induction therapy and has been shown to be non-inferior in achieving remission [14,15]. Current evidence-based management of ANCA-associated vasculitis with renal involvement further emphasizes early induction therapy tailored to disease severity, recommending high-dose glucocorticoids in combination with either cyclophosphamide or rituximab for severe disease, including rapidly progressive glomerulonephritis [20]. These analyses also highlight that advanced chronic damage on kidney biopsy is associated with a low likelihood of renal recovery, regardless of the immunosuppressive regimen used [20]. In the present case, standard induction with cyclophosphamide and high-dose corticosteroids was initiated promptly once the diagnosis was made; however, because of the advanced renal involvement and suboptimal response, B-cell depleting therapy with rituximab was added. Despite these interventions, irreversible kidney damage had already occurred by the time treatment began, and the patient progressed to dialysis-dependent renal failure. This outcome underscores the importance of early diagnosis and intervention in GPA to prevent permanent organ loss. Notably, evidence from other autoimmune diseases suggests that timely use of targeted biologic therapies can improve disease control and limit irreversible damage [21,22].

This case report has several limitations. First, no clinical photographs documenting the orbital involvement were obtained at the time of initial presentation, which limits visual correlation of the dacryoadenitis. Second, quantitative immunophenotyping data were not available, precluding objective assessment of B-cell depletion following rituximab therapy. These limitations do not affect the diagnostic certainty, which was based on serologic findings and definitive renal histopathology.

## 4. Conclusions

This case underscores the clinical diversity of granulomatosis with polyangiitis and the diagnostic challenges associated with atypical presentations. Orbital inflammation, particularly bilateral dacryoadenitis, may serve as an early manifestation of systemic vasculitis and warrants prompt investigation, including ANCA testing and kidney function assessment. Delayed recognition can lead to irreversible renal damage despite aggressive immunosuppression. Early diagnosis, histopathologic confirmation, and timely initiation of appropriate therapy (including biologic agents such as rituximab) are key to improving outcomes in GPA.

## Figures and Tables

**Table 1:** Key laboratory findings in February 2025.

Parameter	Patient’s Value (Normal Range)
Hemoglobin	92 g/L (normocytic, normochromic; normal 120–160 g/L)
C-reactive protein (CRP)	48.8 mg/L (elevated; normal <5 mg/L)
Serum creatinine	516 µmol/L (elevated; normal 45–90 µmol/L)
ANCA (indirect IF)	c-ANCA positive (perinuclear ANCA negative)
Anti-PR3 ELISA	91 U/mL (high positive; normal <5 U/mL)
Anti-MPO ELISA	Negative (normal <5 U/mL)
Urinalysis—hematuria	3+ blood (dysmorphic RBC ~2215/µL)
Urinalysis—proteinuria	2+ protein (24-h protein 2.0 g/day)

## Data Availability

No new datasets were generated or analyzed in this study. Data supporting the findings of this report are available from the corresponding author upon reasonable request.

## References

[1] Jennette J.C., Falk R.J., Bacon P.A., Basu N., Cid M.C., Ferrario F., Flores-Suarez L.F., Gross W.L., Guillevin L., Hagen E.C. (2013). 2012 Revised International Chapel Hill Consensus Conference Nomenclature of Vasculitides. Arthritis Rheum..

[2] Comarmond C., Cacoub P. (2014). Granulomatosis with polyangiitis (Wegener): Clinical aspects and treatment. Autoimmun. Rev..

[3] Hoffman G.S., Kerr G.S., Leavitt R.Y., Hallahan C.W., Lebovics R.S., Travis W.D., Rottem M., Fauci A.S. (1992). Wegener granulomatosis: An analysis of 158 patients. Ann. Intern. Med..

[4] McCluskey R.T., Fienberg R. (1983). The pathogenesis of Wegener’s granulomatosis. Hum. Pathol..

[5] Bullen C.L., Liesegang T.J., McDonald T.J., DeRemee R.A. (1983). Ocular complications of Wegener’s granulomatosis. Ophthalmology.

[6] Haynes B.F., Fauci A.S. (2022). Disorders of vasculature and connective tissue. Harrison’s Principles of Internal Medicine.

[7] Pakrou N., Selva D., Leibovitch I. (2006). Wegener’s granulomatosis: Ophthalmic manifestations and management. Curr. Opin. Ophthalmol..

[8] Hogan S.L., Nachman P.H., Wilkman A.S., Jennette J.C., Falk R.J. (1996). Prognostic markers in patients with antineutrophil cytoplasmic antibody-associated microscopic polyangiitis and glomerulonephritis. J. Am. Soc. Nephrol..

[9] Flossmann O., Berden A., de Groot K., Hagen C., Harper L., Heijl C., Höglund P., Jayne D., Luqmani R., Mahr A. (2011). Long-term patient survival in ANCA-associated vasculitis. Ann. Rheum. Dis..

[10] Little M.A., Nightingale P., Verburgh C.A., Hauser T., De Groot K., Savage C., Jayne D., Harper L. (2010). Early mortality in systemic vasculitis: Relative contribution of adverse events and active vasculitis. Ann. Rheum. Dis..

[11] de Lind van Wijngaarden R.A., Hauer H.A., Wolterbeek R., Jayne D.R., Gaskin G., Rasmussen N., Ferrario F., Hagen E.C., Bruijn J.A., Bajema I.M. (2006). Clinical and histologic determinants of renal outcome in ANCA-associated vasculitis: A prospective analysis. J. Am. Soc. Nephrol..

[12] Booth A.D., Almond M.K., Burns A., Ellis P., Gaskin G., Neild G.H., Plaisance M., Pusey C.D., Jayne D.R.W. (2003). Outcome of ANCA-associated renal vasculitis: A 5-year retrospective study. Am. J. Kidney Dis..

[13] Berden A.E., Ferrario F., Hagen E.C., Jayne D.R., Jennette J.C., Joh K., Neumann I., Noël L.-H., Pusey C.D., Waldherr R. (2010). Histopathologic classification of ANCA-associated glomerulonephritis. J. Am. Soc. Nephrol..

[14] Stone J.H., Merkel P.A., Spiera R., Seo P., Langford C.A., Hoffman G.S., Kallenberg C.G., Clair E.W.S., Turkiewicz A., Tchao N.K. (2010). Rituximab versus cyclophosphamide for ANCA-associated vasculitis. N. Engl. J. Med..

[15] Jones R.B., Tervaert J.W.C., Hauser T., Luqmani R., Morgan M.D., Peh C.A., Savage C.O., Segelmark M., Tesar V., van Paassen P. (2010). Rituximab versus cyclophosphamide in ANCA-associated vasculitis. N. Engl. J. Med..

[16] Westman K.W., Bygren P.G., Olsson H., Ranstam J., Wieslander J. (1998). Relapse rate, renal survival, and cancer morbidity in Wegener’s granulomatosis. Nephrol. Dial. Transpl..

[17] Guillevin L., Pagnoux C., Karras A., Khouatra C., Aumaître O., Cohen P., Maurier F., Decaux O., Ninet J., Gobert P. (2014). Rituximab versus azathioprine for maintenance in ANCA-associated vasculitis. N. Engl. J. Med..

[18] Lopes Caçola R., Alves Morais S., Carvalho R., Môço R. (2016). Bilateral dacryoadenitis as initial presentation of a locally aggressive and unresponsive limited form of orbital granulomatosis with polyangiitis. BMJ Case Rep..

[19] Kawajiri T., Iwata S., Tanaka K., Sonoda T., Nishikawa M., Iwamoto R., Takahashi Y., Kojima F., Fujii T. (2025). Granulomatosis with polyangiitis with lacrimal gland enlargement and pancreatic swelling: A case report and a literature review. Mod. Rheumatol. Case Rep..

[20] Watanabe R., Oshima M., Nishioka N., Sada K.-E., Nagasaka K., Akiyama M., Ando T., Higuchi T., Inoue Y., Kida T. (2023). Systematic review and meta-analysis for 2023 clinical practice guidelines of the Japan Research Committee of the Ministry of Health, Labour, and Welfare for Intractable Vasculitis for the management of ANCA-associated vasculitis. Mod. Rheumatol..

[21] Dimitrov S., Hristova S., Bogdanova-Petrova S., Shivacheva T. (2020). Treatment of systemic lupus erythematosus patients with belimumab–prospective observation over 2 years. Ann. Rheum. Dis..

[22] Dimitrov S., Hristova S., Bogdanova-Petrova S., Shivacheva T. (2012). Impact of treatment with adalimumab on disease activity, work productivity and workday loss in patients with ankylosing spondylitis. Ann. Rheum. Dis..

